# Hypogammaglobulinemia and Poor Performance Status are Predisposing Factors for Vancomycin-Resistant Enterococcus Colonization in Patients with Hematological Malignancies

**DOI:** 10.4274/tjh.2016.0108

**Published:** 2017-03-01

**Authors:** Elif Gülsüm Ümit, Figen Kuloğlu, Ahmet Muzaffer Demir

**Affiliations:** 1 Trakya University Faculty of Medicine, Department of Hematology, Edirne, Turkey; 2 Trakya University Faculty of Medicine, Department of Infectious Diseases, Edirne, Turkey

**Keywords:** Hypogammaglobulinemia, Leukemia, Lymphoma, Myeloma, Vancomycin-resistant Enterococcus

## Abstract

**Objective:**

Vancomycin-resistant enterococci (VRE) are common pathogens of hospital-acquired infection. Long hospitalization periods, use of broad-spectrum antibiotics, and immunosuppression are major risks for VRE colonization. We aimed to evaluate patients’ characteristics and factors that may contribute to VRE colonization.

**Materials and Methods:**

Data of 66 patients with colonization and 112 patients without colonization who were hospitalized in the hematology clinic were collected. Hematological malignancies, preexisting gastrointestinal complaints, the presence of hypogammaglobulinemia at the time of diagnosis, complications like neutropenic enterocolitis (NEC), and Eastern Cooperative Oncology Group (ECOG) and Karnofsky performance statuses were recorded.

**Results:**

Ages of the patients ranged between 19 and 95 years (mean: 55.99). Karnofsky and ECOG scores were statistically related to VRE colonization (p<0.000 and p<0.000), though only the Karnofsky score was significant based on logistic regression analysis. Almost all patients with acute leukemia (45 patients) had been on antibiotics (piperacillin-tazobactam, ceftazidime, and meropenem), while no patients with myelodysplastic syndrome, myeloma, or benign diseases and 2 patients with lymphoma and 1 with chronic myeloid leukemia were on antibiotics. Median time for colonization regardless of antibiotic use and diagnosis was 4.5 days (range: 3-11 days). In the VRE-colonized group, 40.9% of patients had NEC development, while in the non-colonized group, only 1.7% had NEC development. In the VRE-colonized group 46 patients (69.7%) and in the non-colonized group 27 patients (24.1%) had hypogammaglobulinemia at diagnosis; among these patients, 23 patients in the VRE-colonized group (50%) had a B-cell malignancy (lymphoma, myeloma, or chronic lymphocytic leukemia).

**Conclusion:**

Besides already anticipated diseases like leukemia, B-cell malignancies are also at high risk for colonization. This proclivity may be attributed to lack of gastrointestinal IgA due to hypogammaglobulinemia. Prolonged hospitalization (>7 days) may also be accepted as a risk factor, independent of diagnosis or antibiotic use. Performance status is also an important factor for colonization, which may be related to poorer hygiene and increased external help.

## Abstract (tr)

**Amaç:**

Vankomisin dirençli enterokok (VRE), sıklığı giderek artan ve tedavisi güç olan hastane kökenli enfeksiyon etkenlerindendir. Hastanede yatmak, geniş spektrumlu antibiyotiklerin kullanılması ve immünsüpresyon varlığı VRE kolonizasyonu için majör risk faktörlerindendir. Çalışmamızda VRE kolonizasyonu için önemli olabilecek risk faktörü ve hastaların özellikleri ile kolonizasyon arasındaki ilişkileri değerlendirmeyi amaçladık.

**Gereç ve Yöntemler:**

Ağustos 2012 ile Ağustos 2015 tarihleri arasında fakültemiz hematoloji kliniğinde yatan ve VRE anal kolonizasyonu saptanan 66 hasta çalışmaya alındı. VRE açısından düzenli sürveyans yapılmakta olduğundan VRE pozitifliği saptanan hastaların demografik verileri ve birincil hastalıklarına ilişkin veriler dosyalarından kayıt edildi.

**Bulgular:**

Hastalar 19-95 yaş aralığında idi (ortalama: 55,99). Karnofsky ve Doğu Kooperatifi Onkoloji Grubu skorları VRE kolonizasyonu ile ilişkili bulundu (p<0,000 ve p<0,000), ancak yalnızca Karnofsky skoru lojistik regresyon analizi ile istatitiksel anlamlılık göstermekte idi. Akut lösemili hastaların hemen tümü antibiyotik altında iken (piperasilin-tazobaktam, seftazidim ve meropenem), miyelodisplastik sendrom, miyelom ya da selim hematolojik hastalar ile 2 lenfoma ve 1 kronik miyeloid lösemi hastası antibiyotik almakta idi. Antibiyotik kullanımı ve tanıdan bağımsız olarak ortalama kolonizasyon süresi 4,5 gün (3-11 gün) idi. VRE kolonize grupta %40,9 hastada nötropenik enterokolit gelişimi gözlenir iken kolonize olmayan hastaların yalnızca %1,7’sinde nötropenik enterokolit geliştiği gözlendi. VRE kolonize hastalardan 46’sında (%69,7) ve kolonize olmayan hastaların 27’sinde (%24,1) tanı sırasında hipogamaglobulinemi var iken bu hastalarda VRE kolonize grubun %50’sinin B hücre kökenli malinitesi olduğu gözlendi (lenfoma, miyelom ya da kronik lenfositik lösemi).

**Sonuç:**

VRE kolonizasyonu, hematoloji/onkoloji kliniklerinde hızlı yayılan ve kontrol altına alınması güç bir problemdir. Hastaneye yatış sayısından ziyade, tanı sırasında hipogamaglobulinemi bulunması, performansın düşük olması ve remisyona girmemek kolonize olmaya ilişkin risk faktörüdür. Hematolojik maliniteler ve tedavileri seyrinde nötropenik eterokolit ve diyare gibi ciddi ve hayatı tehdit edici komplikasyonlar VRE kolonize olan hastalarda daha sıklıkla gözlenmiştir.

## INTRODUCTION

Vancomycin-resistant enterococci (VRE) cause colonization or infection, especially in immunocompromised patients. Risk factors of VRE colonization include long periods of hospital stay, socioeconomic status, use of broad-spectrum antibiotics, and immunosuppression (neutropenia or immunosuppressive therapy) [1,2]. Since the treatment options for VRE infections are very limited, building up effective approaches to prevent VRE colonization is vital. Intensified infection-control measures like mandatory alcohol-based hand sanitation and use of disposable gloves and gowns, patient cohorting and isolation of colonized patients thorough VRE screening (for rectal colonization), and regular training of the staff and patients are crucial.

First described in the 1980s, the prevalence of VRE infections increased from 4.6 to 9.4 hospitalizations per 100,000 population in 2003-2006 [3]. Related to adverse outcomes, mortality is significantly higher in infections with resistant isolates [1].

From this point of view, we aimed to evaluate additional risk factors for VRE colonization in patients with hematological malignancies and contribute our perspective.

## MATERIALS AND METHODS

Trakya University Hospital is a 1042-bed tertiary-care teaching hospital in Edirne with an annual inpatient admission of 23,000. The annual rate of hospitalization in the hematology inpatient clinic is 550-600. The first case of VRE colonization in our hospital was recognized in 2007 in the intensive care unit. We reviewed all cases of VRE colonization in the hematology inpatient clinic between 2011 and 2014. Systematic surveillance was performed in our hospital for the detection of VRE colonization. Routine swabs for cultures were obtained from the rectum of all patients on admission and twice weekly until discharge. Colonization of VRE was defined as positive results at any time during hospitalization. Decolonization or a negative test was defined as two consecutive negative cultures.

Medical records of patients were reviewed and data regarding age, sex, primary diagnosis for hospitalization, the presence of hypogammaglobulinemia at the time of diagnosis, total leukocyte count, Eastern Cooperative Oncology Group (ECOG) [4] and Karnofsky performance statuses [5] at the time of hospitalization, previous antibiotic use, and history of gastrointestinal complaints were recorded.

Statistical analysis was performed with IBM SPSS Software. Categorical variables were compared using chi-square and Fisher exact tests while non-parametric variables were analyzed with the Mann-Whitney U test. Logistic regression was performed for all significant values and p-values <0.05 were accepted as significant.

Approval from the local ethics committee was obtained for this retrospective study.

## RESULTS

Ages of the patients ranged between 19 and 95 years (mean: 55.99) and 78 patients were female (43.8%) while 100 were male (56.2%). Thirty-seven patients (20.8%) had acute myeloid leukemia (AML), 12 (6.7%) had acute lymphoblastic leukemia (ALL), 48 (27%) had lymphoma, 27 (15.2%) had myeloma, 18 (10.1%) had chronic lymphocytic leukemia (CLL), 5 (2.8%) had chronic myeloid leukemia (CML), 19 (10.7%) had myelodysplastic syndrome (MDS), and 12 patients (6.7%) had a benign hematological disease (immune thrombocytopenia or autoimmune hemolytic anemia). Almost all patients with acute leukemia (45 patients, 68%) had been using antibiotics for febrile neutropenia (23 patients piperacillin-tazobactam, 21 ceftazidime, and 1 meropenem). Median time for colonization, from the first day of antibiotic use to determination of colonization, was 7 days (mean: 4-11). Only 2 patients with lymphoma and 1 CML patient were on antibiotics (ampicillin-sulbactam for non-neutropenic fever) and time for colonization after the use of antibiotics was >7 days. Patients with MDS, myeloma, and benign hematologic diseases with colonization were not taking antibiotics. Mean time from hospitalization (and also a hematological diagnosis) to colonization was 8.5 days (3-14 days).

Performance statuses of the patients were evaluated by the ECOG and Karnofsky performance systems. Forty-six of the VRE-colonized patients (69.66%) had ECOG scores of 3 or 4 while 25 of the not-colonized group (22%) had ECOG scores of 3 or 4 (p<0.005). Fifty patients (83.3%) in the VRE-colonized group and 16 patients in the not-colonized group (14.2%) had Karnofsky performance scores of 40% or lower (p<0.005).

According to the initial complaints, 28 patients in the VRE-colonized group (42.42%) and 5 in the not-colonized group (4.46%) had preexisting gastrointestinal problems (diarrhea, constipation, irritable bowel syndrome) (p<0.005). Within the whole group, neutropenic enterocolitis (NEC) developed in 29 patients (16.29%). In the VRE-colonized group, 40.9% had NEC development, while in the not-colonized group, only 1.7% had NEC development (p<0.005).

In the VRE-colonized group 46 patients (69.7%) and in the not-colonized group 27 patients (24.1%) had hypogammaglobulinemia at the time of diagnosis (p<0.005). Among VRE-positive patients with hypogammaglobulinemia, 23 patients in the VRE-colonized group (50%) had B-cell lineage malignancies (lymphoma, myeloma, and CLL).

Within the whole group, 88 patients were observed to not be in remission (49.4%). Among VRE-positive patients, 46 patients were not in remission (69.69%), while among the not-colonized group, 42 patients (37.5%) were not in remission (p<0.000). Two patients died of causes related to VRE infection (3%). Characteristics of the patients are summarized in Table 1.

With logistic regression analysis, odds ratios for hypogammaglobulinemia, NEC development, preexisting gastrointestinal complaints, ECOG and Karnofsky performance scores, and remission status were 4.62, 39.35, 2.31, 1.7, 29.0, and 1.8, respectively, suggesting a strong relation with hypogammaglobulinemia, NEC development, and Karnofsky performance scores. Data regarding statistical analysis are summarized in Table 2.

## DISCUSSION

Infection with VRE typically follows colonization, mainly of the gastrointestinal tract. Colonization may last for a long period without causing any symptoms and may play a role like a reservoir for the transmission of VRE from one patient to another. Active surveillance for VRE in high-risk clinics such as intensive care units, transplantation units, and hematology-oncology clinics is crucial for preventing infections and further transmission. Risk factors of patients for VRE colonization are well described as being immunocompromised or receiving multiple and prolonged courses of antibiotics and cytotoxic treatments, which all cause impairment of the gastrointestinal mucosa, gastrointestinal flora, and systemic immune system [6]. In our study, especially in patients with leukemia, antibiotic use was significant for colonization, though the duration of hospitalization was not as significant as expected.

Most patients with VRE colonization will remain colonized for long periods. Spontaneous decolonization occurs infrequently and there is no treatment to eliminate colonization. Antimicrobials like oral bacitracin, novobiocin, and ramoplanin are reported to have limited effects in eliminating VRE [7,8]. Since eliminating and eradicating VRE is almost impossible once colonization occurs, controlling and preventing transmission should be the main goal of surveillance. VRE may survive (>1 week) in the environment, can be transferred by hands, and may be isolated from almost every object in health care facilities. Maintaining infection control measures is vital, such as educating staff and patients to use single-use disposable gloves and gowns and frequent hand sanitation. A more detailed list of control measures is available from the Centers for Disease Control and Prevention [9].

Besides the known risk factors, hypogammaglobulinemia is a distinct state of immunodeficiency, with various causes and manifestations and complications. A common and important clinical consequence of hypogammaglobulinemia is predisposition toward infections that are otherwise prevented by antibody-related immune responses (including encapsulated bacteria *Streptococcus pneumoniae* and *Haemophilus influenzae*). Acquired or secondary major causes of hypogammaglobulinemia include drugs, renal and gastrointestinal protein loss, B-cell-related malignancies, and severe burns. The majority of renal diseases leading to hypogammaglobulinemia are nephrotic syndrome, where IgG is lost accompanied by albumin. Gastrointestinal conditions include protein-losing enteropathy and intestinal lymphangiectasia. The clinical manifestations are related to the type and severity of the immunoglobulin lost. In general, hypogammaglobulinemia results in recurrent infections with encapsulated bacteria primarily localized to the upper or lower airways. An agent used in both lymphoproliferative and rheumatic diseases, rituximab, an anti-CD20 antibody, was recently reported to cause significant hypogammaglobulinemia [10]. In our study, hypogammaglobulinemia was observed in 69.7% of the patients at the time of diagnosis. In hypogammaglobulinemic patients, 50% had a B-cell-related lineage malignancy, such as lymphoma or myeloma. Without the burden of treatment, hypogammaglobulinemia is observed to be an independent risk factor in VRE colonization.

Assessment of performance of a patient brings many potential benefits. First, it helps physicians to document how the disease affects the daily living abilities of a person and to determine appropriate risk-adapted treatment and also predict the prognosis. The most generally used performance scores are the Karnofsky and ECOG scores [4,5]. Performance status is also related to a lack of personal hygiene and requirements for constant assistance. Since colonization of VRE is associated with the caregiver’s use of hospital equipment and the surroundings, the increase of colonization in patients with poorer performance is to be expected.

There are limitations of our study. First of all, the number of patients is small. In a larger patient group, both positive and negative colonization groups may demonstrate more credible results. The second limitation is the lack of globulin quantitation. Since the study was not designed as a prospective study in the first place, quantitative immunoglobulin analysis was not performed. Finally, the most important limitation may be the study design. A prospective observational study with a large number of patients is needed to assess our findings.

## CONCLUSION

In clinics dealing with patients with VRE colonization, isolation of the patient as well as related materials, the extra work of disinfecting, active surveillance, and repeated education of staff complicates the management, both economically and socially. Prevention of colonization must be the first goal of all hospitals.

## Figures and Tables

**Table 1:**
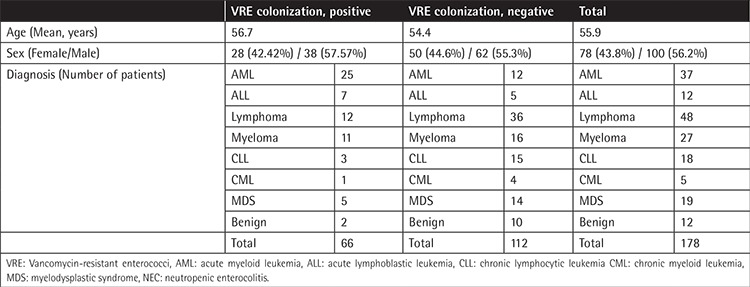
General features of the patients with vancomycin-resistant *Enterococcus* colonization.

**Table 2:**
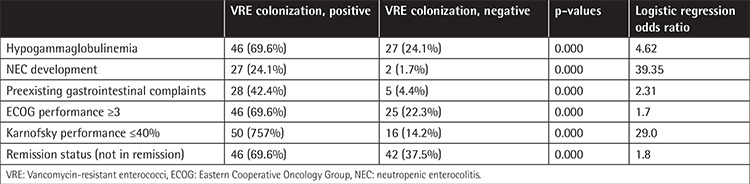
Performance status, hypogammaglobulinemia, and vancomycin-resistant *Enterococcus* colonization.
